# Laparoscopic longitudinal pancreatojejunostomy and modified Frey's operation for chronic calcific pancreatitis

**DOI:** 10.1002/bjs5.50185

**Published:** 2019-06-24

**Authors:** P. Senthilnathan, N. Subrahmaneswara Babu, A. Vikram, S. C. Sabnis, S. Srivatsan Gurumurthy, N. Anand Vijai, V. P. Nalankilli, C. Palanivelu

**Affiliations:** ^1^ Department of Surgical Gastroenterology and HPB Surgery GEM Hospital and Research Centre, 45/A, Pankaja Mill Road, Ramanathapuram, Coimbatore Tamil Nadu–641045 India

## Abstract

**Background:**

Chronic pancreatitis is a debilitating disease presenting with pain, diabetes and steatorrhoea. Surgery offers better long‐term pain relief than other interventions, but there is still uncertainty about the optimal surgical procedure and approach and a lack of long‐term follow‐up data in patients with chronic calcific pancreatitis selected for laparoscopic surgical treatment.

**Methods:**

This was an observational cohort study of patients who underwent laparoscopic surgery for chronic calcific pancreatitis between January 2006 and April 2017, and had completed a minimum follow‐up of 1 year at a tertiary‐care teaching institute. Eligibility for the laparoscopic approach was main duct diameter greater than 7 mm, absence of extensive head calcification, size of head less than 3·5 cm, absence of local complications, and ASA grade I or II status. The primary outcome variable was a reduction in pain score by 1 year. Secondary outcomes were hospital stay, complications, pain score at 3 and 5 years, and the development or progression of exocrine and endocrine insufficiency.

**Results:**

Some 57 patients were scheduled to undergo laparoscopic surgery for chronic pancreatitis: longitudinal pancreatojejunostomy (39), modified Frey's procedure (15) and pancreatoduodenectomy for suspicion of malignancy (3). The latter three patients were excluded from the analysis. Conversion to open surgery was needed in ten of the 57 patients (18 per cent). The mean(s.d.) age of the analysed cohort was 34·2(3·7) years and there was a predominance of men (34, 63 per cent). Adequate pain relief was achieved in 91, 89 and 88 per cent of patients at 1, 3 and 5 years of follow‐up respectively.

**Conclusion:**

Laparoscopic surgical management of chronic calcific pancreatitis with longitudinal pancreatojejunostomy or modified Frey's procedure is feasible, safe and effective in selected patients for the relief of pain.

## Introduction

Chronic calcific pancreatitis (CCP) is a debilitating disease characterized by recurrent abdominal pain, with gradual loss of gland function leading to exocrine and endocrine failure with steatorrhoea and diabetes. Traditionally, chronic pancreatitis is managed with dietary modifications, pancreatic supplements and analgesia. Invasive interventions are reserved for selected patients with intractable pain or those with local complications. Surgery provides better long‐term pain relief than endoscopic interventions^1^. Longitudinal pancreatojejunostomy (Puestow procedure) or with the addition of partial resection of the pancreatic head (Frey's procedure) is the most frequently performed operation for chronic pancreatitis worldwide.

Laparoscopy in the surgical management of chronic pancreatitis is technically challenging. Anatomical landmarks may be difficult to identify as a result of recurrent episodes of inflammation. Tortuosity of the main pancreatic duct, potential for heavy bleeding, and an extended intracorporeal anastomosis can also pose difficulties.

There is limited literature evaluating minimally invasive surgery for this disorder, with a lack of long‐term data regarding pain control and gland function^2^, ^3^. This may become increasingly relevant given emerging reports suggesting a role for early surgery in managing chronic pancreatitis for better pain control and preserved gland function^4^.

This study reports a long‐term experience in the management of CCP using laparoscopic drainage procedures.

## Methods

This study involved a cohort of patients who underwent laparoscopic surgical interventions for unremitting abdominal pain related to CCP between January 2006 and April 2017. Patients whose operations were converted to open procedures were included, except those who underwent head resections for suspected malignant lesions.

GEM Hospital and Research Centre is a tertiary‐care teaching hospital that serves a local population of 2·5 million, with additional referrals from nearby institutions. The annual volume of pancreatic surgeries ranges between 110 and 120 procedures. All operations were performed by two senior authors, with minimum experience of more than 50 laparoscopic pancreatic resections.

### Patient selection

Surgical decompression was reserved for patients with intractable pain requiring regular use of opiate analgesia or who had developed local complications. The laparoscopic approach was offered to those with a main duct diameter greater than 7 mm, absence of extensive head calcifications or mass formation within the head of the gland, and a head size of less than 3·5 cm (maximum head diameter in any direction) on contrast‐enhanced CT, and ASA grade I or II disease. The choice between longitudinal pancreatojejunostomy and a modified Frey's procedure was based on the extent of involvement of the pancreatic head. Patients thought to have predominantly head disease were selected for a modified Frey's operation. Until 2008, only longitudinal drainage was performed by laparoscopy, with the gradual introduction of laparoscopic modified Frey's procedure in selected patients after this date. The terminology and description of the modified Frey's procedure used here has been described previously^5^.

### Data collection

The records were obtained from a prospectively developed database for patients undergoing surgical treatment for chronic pancreatitis. Clinicopathological data, operative variables, postoperative morbidity and length of hospital stay were recorded. During follow‐up visits, pain scores, types of pain relief (complete or partial), and new onset of pancreatic exocrine and endocrine insufficiency were noted and analysed. After discharge, follow‐up visits were scheduled for 7 days, 3 months and 1 year after surgery, then annually. Personal visits were supplemented by telephone interviews.

### Definitions

Pancreatic endocrine insufficiency was diagnosed when the fasting glucose level was above 7 mmol/l, requiring treatment for glycaemic control. Biochemical tests were not performed routinely to diagnose exocrine insufficiency, which was considered present if the patient had steatorrhoea requiring oral pancreatic enzyme supplementation^6^. Pain was evaluated using the Izbicki pain score, a validated system for chronic pancreatitis^7^. The score ranges from 0 to 100, based on a visual analogue scale, the frequency of pain, need for analgesic medication and disease‐related inability to work. The pain was evaluated using a questionnaire that was completed before surgery and during the follow‐up at 1, 3 and 5 years. Relief of pain after surgery was considered partial (Izbicki score of 10 or more after a decrease of over 50 per cent) or complete (Izbicki score of less than 10). Pancreas‐specific complications were diagnosed using the International Study Group of Pancreatic Surgery definitions^8^, ^9^.

### Surgical technique

**Figure 1:**
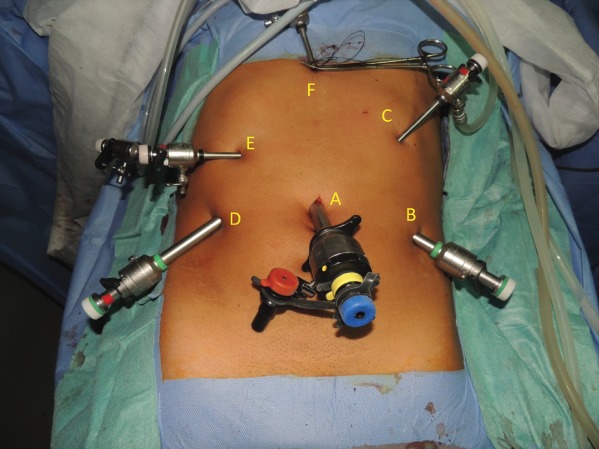
Port positionsA, Umbilical 13‐mm port; B, 10‐mm right‐hand working port; C, 5‐mm port for stomach retraction; D, 10‐mm left‐hand working port; E, 5‐mm left‐hand working port; F, epigastric port for liver and stomach retraction.

**Figure 2:**
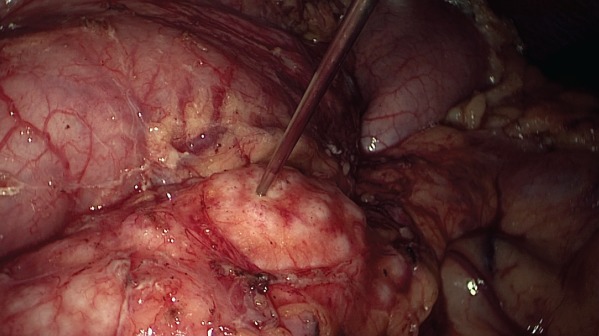
Identification of main pancreatic duct with needle aspiration

**Figure 3:**
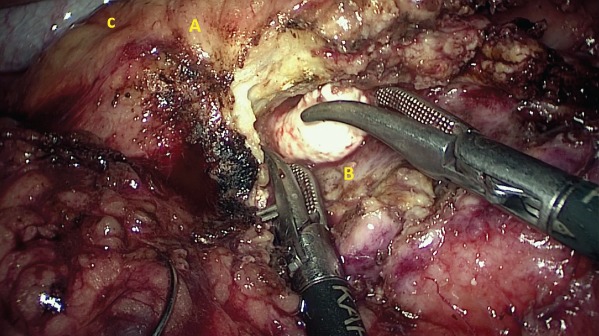
Extraction of stonesA, Pancreatic head; B, Posterior surface of main pancreatic duct; C, C loop of duodenum.

**Figure 4:**
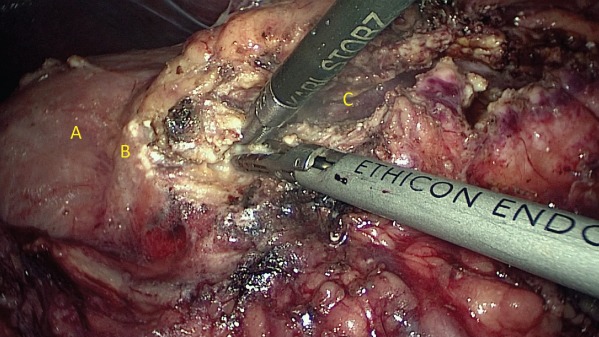
Head coringA, C loop of the duodenum; B, rim of pancreatic head; C, main pancreatic duct laid open.

**Figure 5:**
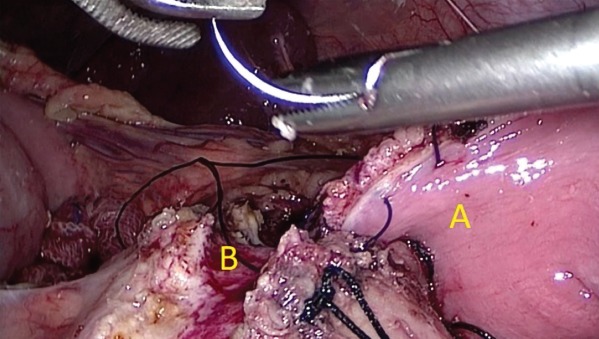
PancreatojejunostomyA, Opened jejunum being anastomosed to main pancreatic duct; B, Opened pancreatic duct.

All patients had bowel preparation the day before surgery. Details of laparoscopic longitudinal pancreatojejunostomy have been described previously^10^. The operative steps until the opening of the pancreatic duct from head to tail were the same in both operations (*Figs* 1 and 2). For those undergoing the modified Frey's procedure, coring out the head of the pancreas was performed by piecemeal excision of pancreatic parenchyma using ultrasonic shears and bipolar coagulation reinforced with haemostatic sutures. The landmark used to determine the extent of head coring was the posterior wall of the main pancreatic duct (*Figs* 3, 4, 5). A single, gravity‐dependent drain was usually placed near the anastomosis for both procedures.

### Postoperative care

Patients were monitored for a minimum of 24 h in a high dependency unit. Oral diet was allowed after the resumption of bowel sounds. On day 3 after surgery, the amylase level in the drain was measured and the drain removed on that day if there was no evidence of a pancreatic leak. Patients were discharged when tolerating solid diet after drain removal.

### Statistical analysis

All continuous variables are reported as mean(s.d.) values. Variables with a non‐normal distribution are reported as median (range) values. Dichotomous variables are expressed as frequencies. Statistical analyses were performed with Excel® (Office) for Windows® version 2007 (Microsoft, Redmond, Washington, USA).

## Results

Between January 2006 and April 2017, 57 patients were offered laparoscopic surgery for CCP, of whom 39 had a longitudinal pancreatojejunostomy and 15 a laparoscopic modified Frey's procedure. Owing to suspicion of malignancy, the remaining three patients had a Whipple procedure and were thus excluded from the final analysis.

The mean age of the 54 patients was 34·2(3·7) years, and the male to female ratio was 1·7 : 1. Most patients were undernourished, with a mean BMI of 24·12(0·67) kg/m^2^, and tropical pancreatitis was the predominant aetiology (56 per cent). All patients had pain, followed by weight loss (83 per cent), steatorrhoea (48 per cent) and diabetes (41 per cent) (*Table* 1).

**Table 1:** Preoperative clinicopathological data

	No. of patients* (*n* = 54)
**Age (years)** †	34·2(3·7)
**Sex ratio (M** : **F)**	34 : 20
**BMI (kg/m** ^**2**^ **)** †	24·12(0·67)
**Aetiology**	
Tropical	30 (56)
Alcoholic	13 (24)
Other	11 (20)
**ASA fitness grade**	
I	25 (46)
II	29 (54)
**Presenting symptoms**	
Abdominal pain	54 (100)
Weight loss	45 (83)
Steatorrhoea	26 (48)
Diabetes mellitus	22 (41)
**Main pancreatic duct diameter on CT (mm)** †	9·7(1·4)

* With percentages in parentheses unless indicated otherwise;

† values are mean(s.d.).

Ten of the initial 57 patients had conversion to open surgery (suspected malignancy in head mass, 3; non‐identification of the pancreatic duct, 3; intraoperative haemorrhage, 4).

The mean duration of surgery for longitudinal pancreatojejunostomy was 220·6(32·0) min and that for the Frey's procedure was 271·0(18·8) min. Mean blood loss in the two groups was 184·5(16·4) and 290·0(34·5) ml respectively. Mean duct diameter was 9·7(1·4) mm. There was a single grade B pancreatic fistula and a single grade A postoperative haemorrhage in each group. Mean duration of hospital was 6·4(1·1) days for longitudinal pancreatojejunostomy and 7·8(1·8) days for the modified Frey's procedure (*Table* 2).

**Table 2:** Intraoperative and postoperative data

	Laparoscopic LPJ (*n* = 39)	Laparoscopic Frey's procedure (*n* = 15)
**Duration of surgery (min)** *	220·6(32·0)	271·0(18·8)
**Operative blood loss (ml)** *	184·5(16·4)	290·0(34·5)
**Length of hospital stay (days)** *	6·4(1·1)	7·8(1·8)
**POPF grade**		
A	2	1
B	1	1
C	0	0
**PPH grade**		
A	1	1
B	0	0
C	0	0
**Follow‐up (months)** *	58·4(20·3)	46·4(16·8)

* Values are mean(s.d.). LPJ, longitudinal pancreatojejunostomy; POPF, postoperative pancreatic fistula; PPH, postpancreatectomy haemorrhage.

Mean follow‐up was 58·4(20·3) months in the longitudinal pancreatojejunostomy group and 46·4(16·8) months in the modified Frey's procedure group. At the end of 1 year, complete pain relief was observed in 49 of the 54 patients (91 per cent). Weight gain was seen in 43 patients (80 per cent). New‐onset endocrine and exocrine insufficiency were found in three of 35 (9 per cent) and two of 24 patients (8 per cent) respectively. After 3 years of follow‐up, complete pain relief was observed in 30 of 34 patients (88 per cent) after longitudinal pancreatojejunostomy and in nine of ten (90 per cent) after modified Frey's procedure. At 5 years of follow‐up, complete pain relief was seen in 21 of 24 patients (88 per cent) after longitudinal pancreatojejunostomy and in seven of eight (88 per cent) after modified Frey's procedure. By this time, new‐onset endocrine insufficiency had risen to 22 per cent (4 of 18) after longitudinal pancreatojejunostomy and 33 per cent (2 of 6) after modified Frey's procedure. New‐onset exocrine insufficiency was seen in 27 per cent (3 of 11) and 33 per cent (2 of 6) respectively at 5 years (*Table* 3).

**Table 3:** Findings at 1, 3 and 5 years of follow‐up

	Total no. of patients	Laparoscopic LPJ	Laparoscopic Frey's procedure
**12 months**	*n* = 54	*n* = 39	*n* = 15
Pain relief	49 (91)	35 (90)	14 (93)
Weight gain (kg)	43 (80)	32 (82)	11 (73)
New‐onset endocrine insufficiency	3 of 35 (9)	2 of 27 (7)	1 of 8 (13)
New‐onset exocrine insufficiency	2 of 24 (8)	1 of 15 (7)	1 of 9 (11)
**36 months**	*n* = 44	*n* = 34	*n* = 10
Pain relief	39 (89)	30 (88)	9 (90)
Weight gain (kg)	36 (82)	28 (82)	8 (80)
New‐onset endocrine insufficiency	5 of 36 (14)	4 of 29 (14)	1 of 7 (14)
New‐onset exocrine insufficiency	4 of 22 (18)	2 of 15 (13)	2 of 7 (29)
**60 months**	*n* = 32	*n* = 24	*n* = 8
Pain relief	28 (88)	21 (88)	7 (88)
Weight gain (kg)	25 (78)	19 (79)	6 (75)
New‐onset endocrine insufficiency	6 of 24 (25)	4 of 18 (22)	2 of 6 (33)
New‐onset exocrine insufficiency	5 of 17 (29)	3 of 11 (27)	2 of 6 (33)

Values in parentheses are percentages. LPJ, longitudinal pancreatojejunostomy.

## Discussion

Experience with laparoscopic surgery for chronic pancreatitis is limited to small case series of longitudinal pancreatojejunostomy and case reports of Frey's procedure^2^, ^3^, ^10^. These studies have, nevertheless, confirmed technical feasibility. Identification of the pancreatic duct is the most critical step and may dictate the need for conversion. In the present series, three patients were converted to open surgery as a result of non‐identification of the duct.

The predominant symptom of pain was relieved in 89 per cent of patients at 3 years, with only a small loss of efficacy at 5 years (88 per cent) with the two operations used. Slightly better pain relief was achieved in the Frey's procedure group at 1 and 3 years. The extent of head coring may influence pain relief, but modification of head coring can give comparable results to deep head coring^5^, ^11^. The posterior surface of the pancreatic duct is the only landmark in laparoscopy to determine the safe limit of head coring, so clearing stones in the branches of the uncinate duct may be a technical limitation in laparoscopy.

New‐onset endocrine insufficiency was observed in four of 18 patients at 5‐year follow‐up after longitudinal pancreatojejunostomy, and in two of six patients in the modified Frey's group, comparable to other studies^11^. Exocrine insufficiency affected 29 per cent of patients, but, despite this, more than three‐quarters had gained weight after surgery after 5 years of follow‐up.

Patients in the present study were generally younger than those undergoing surgery for chronic pancreatitis in the West, with a larger main duct diameter, reflecting aetiology in southern India^12^. Duration of surgery was gradually reduced with the increase in experience, resulting in a mean of 221 min for longitudinal pancreatojejunostomy and 271 min for modified Frey's procedure, comparable to historical open surgery at the authors' institution.

This study is not without limitation. A prospective cohort comprising highly selected individuals, undergoing advanced laparoscopic surgery at a high‐volume centre, with no open comparator arm, means these results should not be considered generalizable. Greater experience gained over the ten years of the study may also have influenced outcomes. Nevertheless, the present study has confirmed the feasibility of the laparoscopic approach with results comparable to those for open surgery, providing substantial pain relief and short hospital stays without compromising the principles of operation for CCP. Further insight into the role of surgery in patients with CCP is likely once the results of the ESCAPE trial^13^ have been published.

## Disclosure

The authors declare no conflict of interest.

## References

[1] Cahen DL , Gouma DJ , Nio Y , Rauws EA , Boermeester MA , Busch OR *et al* Endoscopic *versus* surgical drainage of the pancreatic duct in chronic pancreatitis. N Engl J Med 2007; 356: 676–684. 17301298 10.1056/NEJMoa060610

[2] Biteman BR , Harr JN , Brody F . Laparoscopic Puestow: lateral pancreaticojejunostomy. Surg Endosc 2016; 30: 5624. 27129567 10.1007/s00464-016-4920-z

[3] Tantia O , Jindal MK , Khanna S , Sen B. Laparoscopic lateral pancreaticojejunostomy: our experience of 17 cases. Surg Endosc 2004; 18: 1054–1057. 15156382 10.1007/s00464-003-9210-x

[4] Yang CJ , Bliss LA , Schapira EF , Freedman SD , Ng SC , Windsor JA *et al* Systematic review of early surgery for chronic pancreatitis: impact on pain, pancreatic function, and re‐intervention. J Gastrointest Surg 2014; 18: 1863–1869. 24944153 10.1007/s11605-014-2571-8

[5] Sakata N , Egawa S , Motoi F , Goto M , Matsuno S , Katayose Y *et al* How much of the pancreatic head should we resect in Frey's procedure? Surg Today 2009; 39: 120–127. 19198989 10.1007/s00595-008-3816-5

[6] American Diabetes Association . Clinical practice recommendations 2005. Diabetes Care 2005; 28 **(** Suppl 1): S1–S79. 15618109 10.2337/diacare.28.suppl_1.s1

[7] Bloechle C , Izbicki JR , Knoefel WT , Kuechler T , Broelsch CE . Quality of life in chronic pancreatitis – results after duodenum‐preserving resection of the head of the pancreas. Pancreas 1995; 11: 77–85. 7667246 10.1097/00006676-199507000-00008

[8] Bassi C , Marchegiani G , Dervenis C , Sarr M , Abu Hilal M , Adham M *et al*; International Study Group on Pancreatic Surgery (ISGPS) . The 2016 update of the International Study Group (ISGPS) definition and grading of postoperative pancreatic fistula: 11 years after. Surgery 2017; 161: 584–591. 28040257 10.1016/j.surg.2016.11.014

[9] Wente MN , Veit JA , Bassi C , Dervenis C , Fingerhut A , Gouma DJ *et al* Postpancreatectomy hemorrhage (PPH): an International Study Group of Pancreatic Surgery (ISGPS) definition. Surgery 2007; 142: 20–25. 17629996 10.1016/j.surg.2007.02.001

[10] Palanivelu C , Shetty R , Jani K , Rajan PS , Sendhilkumar K , Parthasarthi R *et al* Laparoscopic lateral pancreaticojejunostomy: a new remedy for an old ailment. Surg Endosc 2006; 20: 458–461. 16424983 10.1007/s00464-005-0680-x

[11] Tan C‐L , Zhang H , Yang M , Li S‐J , Liu X‐B , Li K‐Z . Role of original and modified Frey's procedures in chronic pancreatitis. World J Gastroenterol 2016; 22: 10 415–10 423. 10.3748/wjg.v22.i47.10415 PMC5175254 28058022

[12] Balakrishnan V , Unnikrishnan AG , Thomas V , Choudhuri G , Veeraraju P , Singh SP *et al* Chronic pancreatitis. A prospective nationwide study of 1,086 subjects from India. JOP 2008; 9: 593–600. 18762690

[13] Ahmed Ali U , Issa Y , Bruno MJ , van Goor H , van Santvoort H , Busch ORC *et al*; Dutch Pancreatitis Study Group. *et al*; Early surgery *versus* optimal current step‐up practice for chronic pancreatitis (ESCAPE): design and rationale of a randomized trial. BMC Gastroenterol 2013; 13: 49. 23506415 10.1186/1471-230X-13-49 PMC3610165

